# A Screening Model for Latent Tuberculosis Infection Among Household Contacts of Patients With Tuberculosis Based on the CyTB Test and an Android App: Protocol for a Development and Implementation Study

**DOI:** 10.2196/95534

**Published:** 2026-10-22

**Authors:** Ramesh Kumar Huda, Deepika Yadav, Pooja Chaudhary, Jayvardhan Singh, Laxmikant Mandhana, Manoj Prajapati, Sumit Kalra, Mukti Khetan, Ramesh Kumar Sangwan, Pankaj Bhardwaj

**Affiliations:** 1 ICMR-National Institute for Implementation Research on Non-Communicable Diseases Jodhpur, Rajasthan India; 2 Indian Institute of Technology Jodhpur Jodhpur, Rajasthan India

**Keywords:** latent tuberculosis infection, tuberculosis contact investigation, household contacts, CyTB skin test, digital health, mobile health, screening, implementation research

## Abstract

**Background:**

Latent tuberculosis infection (LTBI) remains a major barrier to tuberculosis elimination, particularly in community settings where household contacts (HHCs) of patients with active tuberculosis are at increased risk of infection and progression to disease. Current tests for LTBI have practical limitations that restrict their use in field and primary care settings. Antigen-based skin testing, combined with digital tools for induration measurement, may improve the accuracy and delivery of HHC screening under routine program conditions.

**Objective:**

This study aims to develop and evaluate a screening model based on CyTB testing and the TBConnect Android app for LTBI among HHCs of patients with confirmed tuberculosis in Rajasthan, India. We will assess its feasibility and operational performance, the prevalence of LTBI among high-risk groups, and implementation outcomes in a routine program setting.

**Methods:**

This protocol describes a community-based interventional implementation study with a mixed-methods design guided by a logic model and the RE-AIM (reach, effectiveness, adoption, implementation, and maintenance) framework, to be conducted over 24 months in the Balesar block, Jodhpur district, Rajasthan. All eligible HHCs of patients with confirmed tuberculosis identified during the study period will be included, with an estimated screening population of 2400 participants. The intervention combines CyTB-based screening with the TBConnect Android app for participant registration, workflow support, induration recording, follow-up, and reporting. Screening will be implemented through health facility–based, home-based, and camp-based pathways. App-assisted induration measurement will be compared with manual assessment in a subset of participants to evaluate agreement between the 2 methods. Primary outcomes will include screening coverage, LTBI positivity, concordance between app-assisted and manual readings, and feasibility of the 3 screening pathways. Secondary outcomes will include health care worker proficiency, app usability, data completeness, operational efficiency, participant acceptability, and documentation of referral advice among participants with positive CyTB screening results.

**Results:**

Ethical approval was obtained from the institutional ethics committee of ICMR-NIIRNCD (Indian Council of Medical Research–National Institute for Implementation Research on Non-Communicable Diseases), Jodhpur, Rajasthan, India, on August 7, 2025. Recruitment and CyTB screening began in December 2025 and are scheduled to continue until December 2026. Data cleaning and analysis are planned for after field data collection, and the main findings are expected to be submitted for publication in May 2027.

**Conclusions:**

This protocol describes the implementation and evaluation of a digitally supported CyTB-based LTBI screening model for HHCs of confirmed tuberculosis patients in Rajasthan, India. The study will generate operational evidence on screening coverage, pathway feasibility, agreement between app-assisted and manual induration readings, data completeness, and user acceptability within routine primary health care services.

**International Registered Report Identifier (IRRID):**

DERR1-10.2196/95534

## Introduction

Tuberculosis remains a major public health problem, especially in low- and middle-income countries [1,2]. The ongoing burden of tuberculosis is driven not only by transmission of active disease but also by the large burden of latent tuberculosis infection (LTBI). Global estimates suggest that approximately one-quarter of the world’s population is infected with *Mycobacterium tuberculosis* in a latent state [3,4]. This burden is important for tuberculosis control because reactivation of latent infection continues to drive new active tuberculosis cases, even when transmission is reduced [5,6]. The identification and management of LTBI are central to long-term tuberculosis elimination efforts [7-9].

The risk of progression from LTBI to active tuberculosis is not uniform across populations. While most infected individuals remain asymptomatic throughout life, the lifetime risk of progression from LTBI to active tuberculosis among immunocompetent individuals is commonly estimated at approximately 5%-10%. In contrast, the risk is substantially higher among individuals with HIV infection, malnutrition, diabetes, or other forms of immunosuppression [10]. Children are particularly vulnerable because they are more likely to progress rapidly after primary infection and to develop severe or disseminated forms of disease [11]. Household contacts (HHCs) with recent exposure represent an especially important group for early identification because recent infection is associated with a higher risk of progression to active disease [12]. These patterns make early diagnosis of LTBI especially important in populations with high exposure and increased biological vulnerability, underscoring the need for effective screening models such as the one proposed in this study to support tuberculosis elimination efforts [13].

HHCs with recent exposure represent a high-risk group for early LTBI identification. Prolonged and close exposure to an infectious index case substantially increases the likelihood of household transmission [14]. LTBI prevalence ranges from approximately 26% to over 50% among HHCs in India. Contact investigation is therefore essential for tuberculosis prevention and control [15]. Under the National Tuberculosis Elimination Programme (NTEP), systematic screening of HHCs can support early diagnosis of eligible individuals for preventive therapy and strengthen a prevention-based approach to tuberculosis control [16,17].

Despite its importance, large-scale LTBI screening remains difficult to implement. The 2 commonly used tests for LTBI, the tuberculin skin test (TST) and interferon-gamma release assays (IGRAs), have important limitations. The TST may exhibit reduced specificity in populations vaccinated with Bacillus Calmette-Guérin (BCG) and may yield false-positive results in settings with widespread BCG coverage [18]. IGRAs demonstrate greater specificity in BCG-vaccinated populations but require venipuncture, laboratory infrastructure, trained personnel, and timely specimen processing, which limit their use in community-based and resource-constrained settings [19]. Neither TST nor IGRA can reliably distinguish LTBI from active tuberculosis. Similarly, CyTB is an immunological skin test used to detect *M. tuberculosis* infection and should not be interpreted as a stand-alone test for differentiating latent infection from active disease. Therefore, symptom screening and referral for diagnostic evaluation of presumptive active tuberculosis remain necessary before LTBI classification and further management. In addition, the cost and availability of LTBI testing options may vary across health systems and program settings, which can influence feasibility and scalability [20,21].

CyTB uses recombinant ESAT-6 and CFP-10 antigens specific to *M* tuberculosis and absent from BCG strains and most nontuberculous mycobacteria. This enhances specificity compared with TST in BCG-vaccinated populations while maintaining the simplicity of a skin test [22]. Evidence indicates good concordance between antigen-based skin tests and IGRAs, with acceptable safety in adults, children, and people living with HIV [4,21,23]. CyTB can be administered intradermally without laboratory processing. It offers a potentially practical option for HHC screening in primary care and community settings due to its operational simplicity and compatibility with field-based implementation [24].

This study explores how implementing the CyTB test, integrated with an Android app, can be optimized to improve the early detection of LTBI among high-risk populations. Effective contact screening of LTBI depends not only on diagnostic performance but also on efficient registration, follow-up, documentation, and reporting. The application of digital tools on smartphones can support workflow management, facilitate data capture, and improve documentation and follow-up during HHC screening activities [25,26]. This approach aims to improve operational efficiency and scalability of HHC screening in routine program settings while adhering to data privacy standards and ethical guidelines. Such tools may also reduce documentation errors, support timely follow-up, and improve completeness of field-level records.

Operational evidence from India on this combined approach remains limited. This study was designed to train health care workers at primary health care facilities to effectively use the CyTB test and the Android app to screen and identify latent tuberculosis among HHCs of patients with confirmed tuberculosis in Rajasthan, India. It also intends to codevelop and optimize a screening model that integrates the CyTB test with the Android app to improve the detection of latent tuberculosis in high-risk populations. Additionally, the study will assess the prevalence of latent tuberculosis among high-risk groups and evaluate the acceptance and impact of the TBConnect app in supporting CyTB testing to enhance detection efficiency and diagnostic accuracy. The findings may contribute operational evidence to inform future LTBI screening strategies in routine tuberculosis program settings.

## Methods

### Study Design and Evaluation Approach

This is a community-based interventional implementation study with a mixed methods evaluation design. The intervention being implemented is a CyTB skin test–based LTBI screening model supported by the TBConnect Android app. The model includes identification of eligible HHCs, digital registration, CyTB administration, follow-up for 48-to-72-hour induration reading, app-assisted induration documentation, result recording, and referral of participants with positive screening results for further evaluation.

The study will compare 3 implementation pathways for delivering the same CyTB-TBConnect screening model: health facility–based, home-based, and camp-based screening. These pathways will be compared as alternative service-delivery approaches, not as distinct diagnostic interventions.

The evaluation will include quantitative and qualitative components. Quantitative data will assess screening coverage, completion of follow-up reading, LTBI positivity, agreement between app-assisted and manual induration readings, data completeness, operational efficiency, and documentation of referral advice for CyTB-positive participants. Qualitative data will explore acceptability, usability, barriers, facilitators, and contextual factors affecting implementation from the perspectives of HHCs and health care workers. A logic model will guide the development and optimization phase, and the RE-AIM (reach, effectiveness, adoption, implementation, and maintenance) framework will guide the implementation evaluation [27,28].

### Implementation Success Criteria

Implementation success will be assessed using predefined screening, operational, digital, and referral-related indicators. The model will be considered feasible if eligible HHCs can be identified and enrolled through routine program systems, if a high proportion of enrolled participants complete CyTB administration and 48-to-72-hour induration reading, and if health care workers can use the TBConnect app for registration, documentation, induration capture, and reporting after training.

The main indicators of implementation success will include screening coverage among eligible HHCs, completion of follow-up reading within the required 48-to-72-hour window, LTBI positivity among screened participants, agreement between app-assisted and manual induration readings, completeness of digital records, time required for test administration and reading, documentation of referral advice among CyTB-positive participants, participant acceptability, health care worker acceptability, and feasibility of delivery through the 3 screening pathways.

### Study Setting

The study will be conducted in the Balesar block, Jodhpur district, Rajasthan, India, over 24 months. The intervention will be implemented by health care staff of community health centers (CHCs), primary health centers (PHCs), and subhealth centers/Ayushman Arogya Mandir. The block comprises a CHC, 5 PHCs, and 52 subcenters.

### Study Participants

The study will include HHCs of patients with confirmed tuberculosis who have had close, prolonged exposure to the index case, are aged 1 year or older, and reside within the study area. An HHC will be defined as an individual residing in the same household as the index patient with tuberculosis or sharing the same enclosed living space during the index case’s infectious period. Confirmed tuberculosis index cases will be identified through routine NTEP records and the Nikshay portal maintained at public health facilities in the study block. Frontline health workers, such as community health officers and auxiliary nurse midwives, will participate in screening, data entry, and follow-up activities to support implementation and evaluation.

### Eligibility Criteria

Eligible participants will include HHCs of patients with confirmed tuberculosis residing in the selected study block who are aged 1 year or older and who provide informed consent or assent, as applicable. Individuals who do not provide informed consent, are unavailable for follow-up reading within the 48-to-72-hour window, or have conditions that preclude safe administration or assessment of the skin test will be excluded. They will be documented in accordance with the study workflow and excluded from completed-screening estimates. Health care workers involved in implementation in the study area will be eligible for participation in the training and evaluation components.

### Sample Size

All eligible HHCs or family members of confirmed tuberculosis patients identified within the selected block during the study period will be invited to participate. The implementation will be conducted in 1 block of Jodhpur district, Rajasthan. The projected screening population was estimated using the approximate block population, expected prevalence of active tuberculosis, and average household size.

The selected block has an approximate population of 300,000. Based on an estimated active tuberculosis prevalence of 0.2% [29], the expected number of confirmed tuberculosis patients in the block is approximately 600. Considering an average household size of 4 members, the estimated number of HHCs or family members eligible for screening is approximately 2400. Therefore, the anticipated screening population for this implementation study is 2400 participants. This protocol aims to inform stakeholders about the study design, procedures, and evaluation methods to facilitate successful implementation.

Although the study is not designed as an individually randomized efficacy trial, the projected sample size is considered adequate for the primary implementation objectives. A sample of 2400 participants will allow estimation of LTBI positivity in the screened population with acceptable precision. The projected screening population represents the expected number of eligible HHCs who may be approached during the study period; actual enrollment and screening completion will depend on participant availability, eligibility, consent, and completion of the 48-to-72-hour reading visit.

To validate app-assisted induration measurement, the required sample size was estimated for an agreement analysis using the intraclass correlation coefficient approach described by Walter et al [30]. Assuming a minimum acceptable intraclass correlation coefficient of 0.85, an expected intraclass correlation coefficient of 0.95, a 2-sided significance level of 0.05, 90% power, 2 ratings per participant, and a 10% allowance for noncompletion of paired readings, the required validation sample size was 37 participants. However, this appeared to be a small sample size. Accordingly, in the current protocol, approximately 50% of participants completing the 48-to-72-hour reading will have paired app-assisted and manual scale-based induration measurements recorded in TBConnect. This proportion is expected to provide a validation subset substantially larger than the minimum required sample size.

### Intervention

#### Overview

The intervention is a digitally supported, CyTB-based LTBI screening model for HHCs of patients with confirmed tuberculosis. It combines CyTB skin testing with the TBConnect Android app to support participant registration, screening workflow documentation, 48-to-72-hour follow-up, induration recording, result classification, and referral documentation. This section details how trained health care workers, including community health officers, auxiliary nurse midwives, laboratory technicians, and designated program staff, will deliver the intervention across health facility–based, home-based, and camp-based pathways, ensuring standardized procedures and criteria throughout.

#### CyTB Test

The CyTB test will be used for LTBI screening among HHCs. The test is approved for use in individuals aged 1 year and older and can be administered in field settings without laboratory infrastructure. It will be administered intradermally using the Mantoux technique with 0.1 mL of CyTB containing rdESAT-6 and rCFP-10 antigens. Induration will be assessed 48-72 hours after administration using the smart patch workflow integrated into the app. An induration of 5 mm or greater will be considered positive, and an induration of less than 5 mm will be considered negative. To validate app-assisted induration measurement, approximately 50% of participants who complete the 48-to-72-hour reading will have paired app-assisted and manual, scale-based induration measurements recorded in TBConnect. This does not represent separate allocation to app-assisted or manual reading groups. TBConnect includes fields for both app-assisted measurement and manual measurement within the same participant record. Manual measurement, performed by trained personnel using the standard palpation-and-ruler method, will be used as the reference standard for validating app-assisted measurement.

#### TBConnect App

The TBConnect Android app will support the CyTB screening workflow. It will be used for participant registration, data capture, image-based induration recording, follow-up reminders, reporting, and analytics. The app will support 2 scans: one at the time of test administration and another at 48 to 72 hours to assess induration. Manual entry of induration measurements will also be available to support validation of app-assisted readings. The app is designed to run on Android phones and tablets. TBConnect includes separate fields for app-assisted and manual scale-based induration measurements. The app-assisted component supports image-based documentation of the induration site. In contrast, the manual component allows trained personnel to enter the induration size measured using the standard palpation-and-ruler method. For the validation subset, both values will be recorded within the same participant record and compared to assess agreement, with manual measurement used as the reference standard. The CyTB-TBConnect screening workflow is shown in Figure 1.

**Figure 1:**
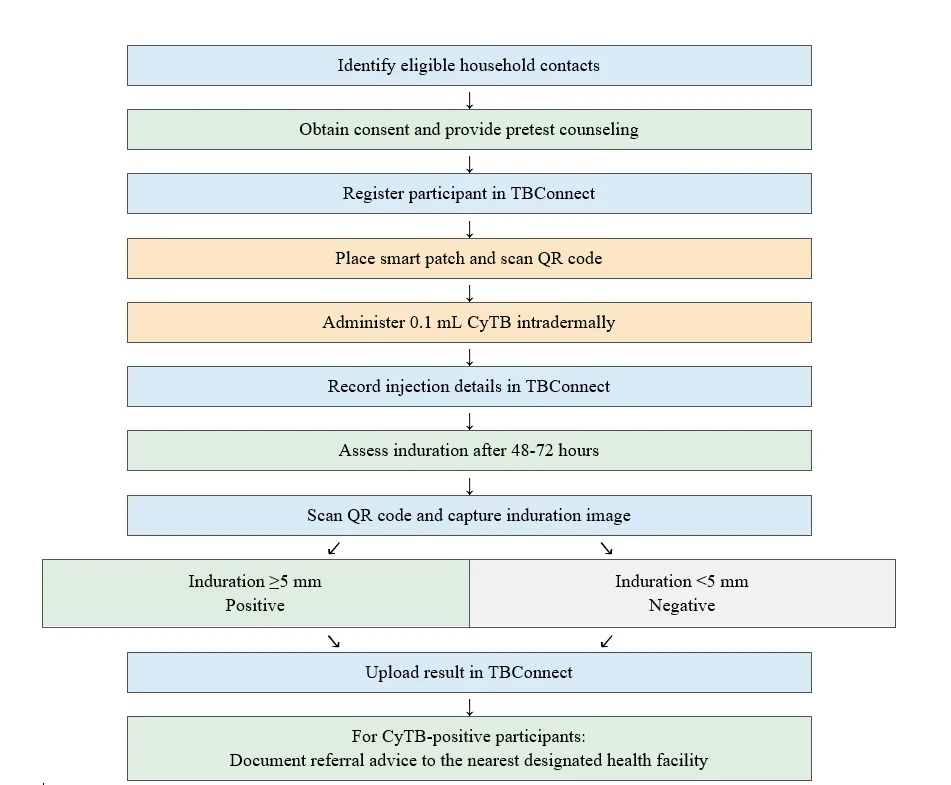
Workflow of CyTB testing and TBConnect-supported screening procedures.

### Screening Procedures

Eligible HHCs will be identified from health facility records documenting patients with confirmed tuberculosis and their household members. Participants meeting the eligibility criteria will be enrolled and registered in the TBConnect app. Trained health care workers will administer CyTB and perform follow-up reading in accordance with standard operating procedures.

Before LTBI testing, participants will be screened for symptoms suggestive of active tuberculosis in accordance with NTEP guidelines, and those with presumptive tuberculosis will be referred for diagnostic evaluation. Following registration, the smart patch will be placed on the participant’s arm, the QR code scanned, the injection site prepared, and the CyTB test administered intradermally using the Mantoux technique. After 48 to 72 hours, app-assisted reading will be performed by scanning the code and capturing the induration image. A predefined subset of participants will additionally undergo manual measurement by trained personnel. For the validation subset, both app-assisted and manual scale-based induration measurements will be obtained during the same 48-to-72-hour reading visit. Manual measurements will be performed by trained personnel using the standard palpation-and-ruler method. To reduce bias, a separate, blinded, trained reader who is unaware of the app-assisted result will independently record the manual measurement. After completing the manual reading, the reader will enter the value into the app. Both measurements will be reflected in TBConnect as continuous values in millimeters and classified as positive or negative using the predefined ≥5 mm threshold.

Participants with positive CyTB screening results will be counseled and advised to attend the nearest designated public health facility for further evaluation and management in accordance with NTEP guidelines. The study will document the positive screening result and referral advice provided. Referral completion, initiation of tuberculosis preventive therapy, adherence, and treatment outcomes will not be assessed as study outcomes. Participants with symptoms suggestive of active tuberculosis at baseline screening will be referred for diagnostic evaluation under routine program procedures. They will not be classified as LTBI-positive based on CyTB screening. The referral advice documented for CyTB-positive participants applies only to individuals with positive LTBI screening results.

### Screening Pathways and Comparison Criteria

Three screening pathways will be evaluated: health facility–based, home-based, and camp-based. In the health facility–based pathway, eligible HHCs will be invited to a participating public health facility for registration, CyTB administration, and follow-up reading. In the home-based pathway, trained health care workers will conduct screening activities at or near the participant’s residence. In the camp-based pathway, screening will be organized through community-level sessions at predefined locations.

### Assignment to Screening Pathways

Participants will not be randomly assigned to the health facility–based, home-based, or camp-based screening pathways. HHCs will be mapped from the list of confirmed active tuberculosis patients obtained from the District Tuberculosis Office, Nikshay portal, and records maintained at participating CHCs, PHCs, and subhealth centers. These records will be used to identify index tuberculosis patients enrolled for treatment and to compile a list of eligible HHCs, including family members and close contacts residing within the same household.

The 3 screening pathways will be implemented within the same operational period during the pilot phase to assess their feasibility under comparable field conditions. Program logistics, location of the index tuberculosis patient, availability of HHCs, field team planning, and local service delivery arrangements will determine pathway allocation. In the health facility–based pathway, HHCs will be invited to the nearest participating health facility for CyTB administration and 48-to-72-hour reading. In the home-based pathway, the field team will visit households directly and assess feasibility based on HHC availability, coverage, CyTB administration, and completion of the second scan. In the camp-based pathway, HHCs will be mobilized to attend predefined community screening sessions.

All HHCs will be informed in advance by telephone or through local health workers about the date and location of the first scan and will be contacted again for the 48-to-72-hour reading. The same CyTB-TBConnect workflow, eligibility criteria, consent procedures, reading window, positivity threshold, and referral advice procedure will be followed across all 3 pathways.

Because pathway allocation is not randomized, comparisons across the 3 pathways will be descriptive and implementation-focused. The findings may be affected by selection bias, differences in participant availability, distance from health facilities, household accessibility, and local program logistics. Therefore, the pathway with the highest HHC coverage and completion of first and second scans will be interpreted as the most feasible approach for further implementation within the study area, rather than as evidence of superior clinical effectiveness.

All 3 pathways will use the same eligibility criteria, consent procedures, CyTB administration method, 48-to-72-hour reading window, positivity threshold, TBConnect documentation process, and referral criteria. Therefore, the pathways will be compared as alternative implementation approaches for delivering the same screening intervention, rather than as different diagnostic interventions.

The 3 pathways are included because HHC screening may require different delivery strategies under routine program conditions. Facility-based screening may allow for closer supervision and easier documentation; home-based screening may reduce access barriers for HHCs; and camp-based screening may improve reach within defined community clusters. The purpose of comparing these pathways is to assess their relative feasibility of implementation, not to determine diagnostic superiority.

The comparison will consider both coverage and operational requirements. Indicators will include the proportion of identified HHCs reached, proportion enrolled, proportion receiving CyTB, proportion completing the 48-to-72-hour reading, LTBI positivity, completeness of TBConnect records, time required for test administration and reading, documentation of referral advice among participants with positive screening results, participant acceptability, health care worker acceptability, staffing requirements, logistical feasibility, and pathway-specific operational challenges.

The pathway with the most favorable implementation performance will not be assumed to be automatically acceptable for wider program use. Its suitability for further implementation will be interpreted in relation to operational feasibility, resource requirements, health system capacity, and local program priorities.

### Study Phases

The study will be implemented in 4 phases. The preparatory phase will include recruitment and training of project staff, procurement of equipment and consumables, stakeholder engagement, pilot-testing of procedures, identification of eligible participants from health records, and deployment of the Android app. The codevelopment and optimization phase will include training block-level staff, piloting the CyTB-TBConnect model, testing the 3 screening pathways, collecting user feedback, and iteratively refining study procedures and the app interface. A logic model will be used in this phase to track inputs, activities, outputs, outcomes, and expected impact (Table 1). During the implementation phase, the optimized model will be deployed across the study block for large-scale screening of HHCs of patients with confirmed tuberculosis, as illustrated in Figure 2. Any modifications to procedures or app features during the optimization phase will be documented systematically. During the evaluation phase, the intervention will be assessed using the RE-AIM framework across the domains of reach, effectiveness, adoption, implementation, and maintenance, as explained in Table 2.

**Table 1:** Logic model for codeveloping and optimizing the screening and identification model phase.

Category and resources		Activities
**Inputs**		
	Resources and materials	Funding and budget allocation CyTB test kits and Android app Training materials and modules Human resources, including health care workers, trainers, and technical support staff Technological resources, including servers, software, and devices for data collection
	Stakeholders	Project staff and health care workers Local health authorities Community leaders Participants (family members of patients with tuberculosis)
**Activities**		
	Training health care workers	Design and develop comprehensive training modules Organize and conduct training workshops for health care workers on the use of the CyTB test and Android app Conduct assessments to certify health care worker proficiency
	Pilot-testing	Conduct a pilot study to test the feasibility of the screening model Implement different screening models (health facility–based, home-based, and camp-based) Collect feedback from health care workers and participants
	Model optimization	Analyze feedback and data from the pilot study Refine the screening protocols and Android app interface Conduct iterative testing and further optimization
**Outputs**		
	Training completion	Number of health care workers trained and certified Training completion rate and assessment scores
	Pilot study data	Data collected from different screening models Feedback from participants and health care workers Initial prevalence rates of latent tuberculosis detected among high-risk participants
**Outcomes**		
	Short-term outcomes	Improved knowledge and skills of health care workers Initial identification of latent tuberculosis cases using the optimized model Increased efficiency and accuracy of latent tuberculosis screening
	Medium-term outcomes	Enhanced usability of the Android app and CyTB test integration
**Impact**		
	Long-term impact	A validated and optimized screening model for latent tuberculosis Increased early detection rates of latent tuberculosis in high-risk populations A scalable model that can be implemented in other regions and settings

**Figure 2:**
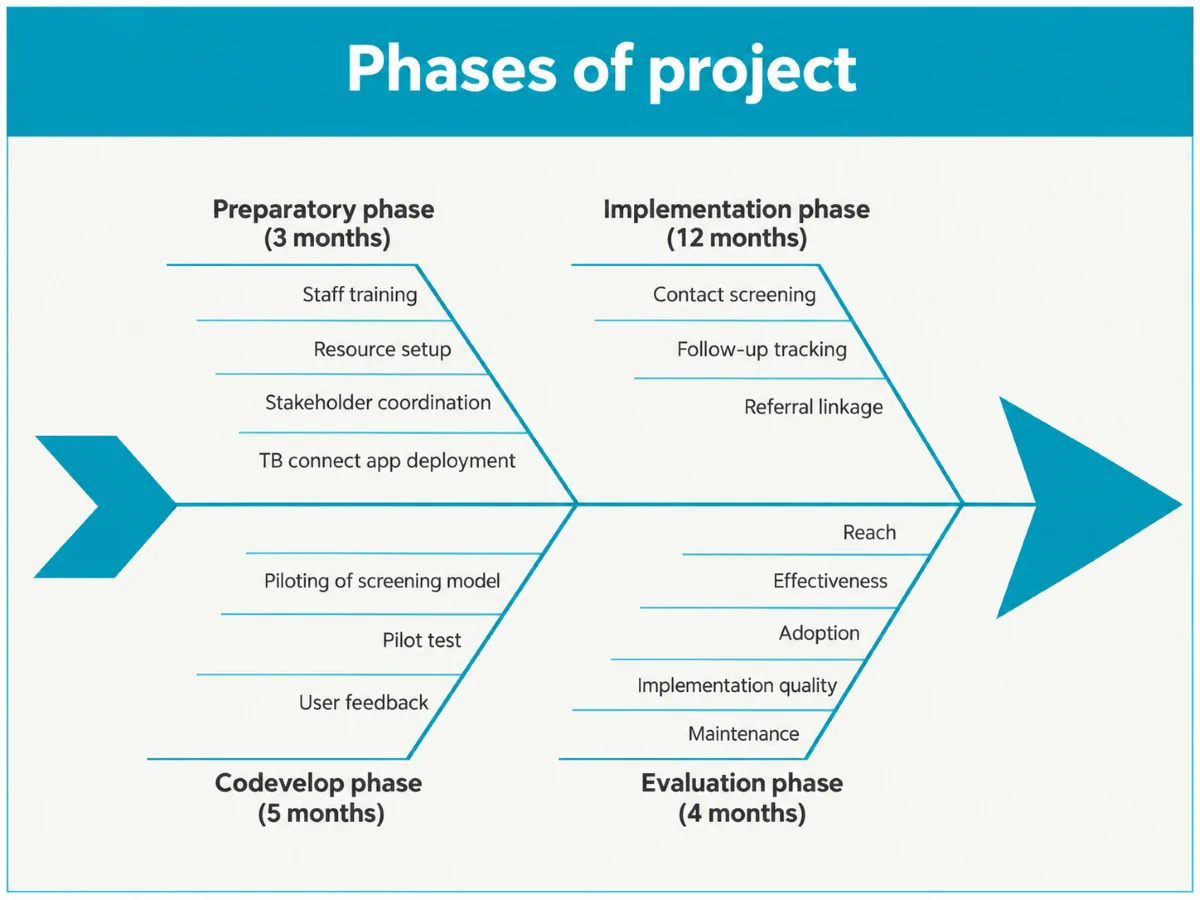
Study phases for development, optimization, implementation, and evaluation of the CyTB-TBConnect screening model.

**Table 2:** Reach, Effectiveness, Adoption, Implementation, and Maintenance framework for the evaluation phase.

Dimension	Objective	Indicators	Data sources/methods
Reach	Participant reach	Screening uptake, participant profile, participant vs nonparticipant differences	TBConnect records, surveys/interviews
Effectiveness	Screening effectiveness	LTBI^a^ positivity, app-assisted vs manual induration, training-related change	Screening data, validation comparison, training assessments, statistical analysis to identify risk factors
Adoption	Staff and setting uptake	Staff trained, usability and acceptability, screening model selection	Training records, surveys/in-depth interviews, analyze the screening model for preferences and challenges
Implementation	Fidelity and quality	Data completeness, timeliness, operational barriers	TBConnect audit, logs, process evaluation
Maintenance	Sustainability	Documentation of referral advice, continued feasibility of app use, potential program integration	TBConnect referral documentation, staff surveys, stakeholder consultation

^a^LTBI: latent tuberculosis infection.

### Structured Reporting of Participant Flow and Outcome Assessment

To improve transparency and reproducibility, participant flow will be documented from the identification of confirmed tuberculosis index cases, listing of eligible HHCs, eligibility assessment, consent or assent, CyTB administration, completion of the 48-to-72-hour reading, validation subset inclusion where applicable, result classification, and documentation of referral advice.

Intervention delivery will be reported separately for health facility–based, home-based, and camp-based pathways. Outcome measures will be grouped into screening, implementation, validation, and acceptability domains. Feasibility will be assessed using HHC coverage, CyTB administration, completion of the second reading, data completeness, time requirements, staffing needs, logistical feasibility, and pathway-specific operational challenges. Acceptability will be assessed through qualitative interviews with HHCs and health care workers.

### Proposed Implementation Strategies

Implementation strategies will include stakeholder engagement, training and capacity building, pilot-testing of screening pathways, iterative model optimization, and community-focused information, education, and communication activities. Social and behavior change communication approaches will be used to address misconceptions, improve community awareness, and support participation in screening and follow-up.

### Data Collection

#### Overview

Data collection will follow a mixed methods approach to evaluate both the performance and implementation of the CyTB-TBConnect screening model. Quantitative data will be collected through the TBConnect app, training records, screening records, validation assessments, and referral documentation. These data will be used to assess reach, screening completion, LTBI positivity, agreement between app-assisted and manual reading, operational efficiency, data completeness, and documentation of referral advice among participants with positive CyTB screening results. Qualitative data will be collected through in-depth interviews with selected HHCs and health care workers to examine acceptability, usability, barriers, facilitators, and contextual factors influencing implementation.

#### Quantitative Data Collection During CyTB Screening

Quantitative data collection will be integrated into the CyTB screening workflow. HHCs of confirmed tuberculosis patients will be identified from routine program records maintained at participating health facilities under the NTEP. Eligibility will be verified based on residence within the study area, age criteria, and exposure to the index case. Eligible HHCs will be enrolled after written informed consent or assent, as applicable, has been obtained. Following enrollment, participants will be registered in the TBConnect Android app, in which baseline information, including demographic characteristics, household details, exposure history, and relevant clinical information, will be recorded.

Before administration of the CyTB test, all participants will be screened for symptoms suggestive of active tuberculosis in accordance with program guidelines. Individuals with presumptive tuberculosis will be referred to the nearest health facility for diagnostic evaluation and will not undergo LTBI testing until active tuberculosis has been excluded. For participants eligible for LTBI screening, the CyTB test will be administered intradermally by trained health care workers using the Mantoux technique. Information related to test administration, including the date and time of injection, testing location, screening pathway (health facility–based, home-based, or camp-based), and identifier of the administering health care worker, will be documented in the TBConnect app.

Participants will be reassessed 48-72 hours after test administration for induration reading. During this follow-up visit, app-assisted induration measurement will be performed using the TBConnect app, and the induration image and associated measurement will be recorded in the digital screening record. In a validation subset comprising approximately 50% of participants who complete the 48-to-72-hour reading, both app-assisted and manual induration measurements will be recorded in TBConnect. Manual measurements will be performed by trained personnel using the standard palpation and ruler method and will serve as the reference standard for validating app-assisted measurements.

Screening outcomes will be recorded as positive or negative according to the predefined induration threshold. For participants with positive CyTB screening results, the study team will document the screening result and the referral advice provided, including the nearest designated health facility recommended for further evaluation in accordance with NTEP guidelines. Postreferral clinical evaluation, initiation of tuberculosis preventive therapy, adherence, and treatment outcomes will not be measured in this implementation evaluation. In addition to screening results, operational and implementation data will be collected throughout the study to assess feasibility and workflow performance. These indicators will include screening completion rates, follow-up reading rates, time required for test administration and reading, completeness of digital records, and pathway-specific operational challenges.

Data related to health care worker training and implementation readiness will also be collected. These will include attendance at training sessions, completion of training modules, certification status, and pre- and posttraining assessment scores evaluating knowledge, attitudes, and practices related to CyTB testing and the use of the TBConnect app.

#### Qualitative Data Collection

Qualitative data will be collected to explore the acceptability, usability, barriers, and facilitators associated with implementing the CyTB-TBConnect screening model. Participants for the qualitative component will be selected through purposive sampling until data saturation is reached, with efforts made to ensure representation across screening pathways and implementation roles. The qualitative sample will include HHCs who participated in screening and health care workers who administered CyTB and used the TBConnect app.

A semistructured interview guide will be developed to explore participants’ and health care providers’ experiences with the screening process, the usability of the TBConnect app, the perceived benefits and challenges of the screening model, and factors influencing follow-up and implementation. In-depth interviews will be conducted by trained research personnel in a private setting to facilitate open discussion. With participant permission, interviews will be audio-recorded and transcribed verbatim. Transcripts will subsequently be reviewed for accuracy and organized for thematic analysis. These qualitative data will complement the quantitative findings by providing a deeper understanding of implementation processes, user experiences, and contextual factors influencing the adoption of the screening model.

### Outcomes

The evaluation will assess screening, implementation, digital workflow, validation, and referral-documentation outcomes associated with the CyTB-TBConnect screening model. Implementation success will be assessed using predefined indicators, including screening coverage among eligible HHCs, completion of CyTB administration, completion of 48-to-72-hour induration reading, LTBI positivity, agreement between app-assisted and manual induration measurements, completeness of TBConnect records, operational feasibility across the 3 pathways, documentation of referral advice among participants with positive results, participant acceptability, and health care worker acceptability described in Table 2.

### Primary Outcomes

Primary outcomes include screening coverage among eligible HHCs, defined as the proportion of identified contacts who complete CyTB screening; LTBI positivity among screened participants; and agreement between app-assisted and manual induration measurements in the validation subset. Feasibility of each pathway will be assessed using predefined indicators, including screening uptake, completion of the 48-to-72-hour reading, data completeness, time required for screening and reading, documentation of referral advice, participant acceptability, and health care worker acceptability.

### Secondary Outcomes

Secondary outcomes will focus on implementation performance and user experience. These will include health care worker proficiency following training, adoption and usability of the TBConnect app among health workers, completeness and accuracy of digital data capture within the app, operational efficiency measured by the time required for test administration and follow-up reading, participant acceptability of the screening process, and documentation of referral advice among participants with positive CyTB screening results. Referral completion, initiation of preventive therapy, adherence, and treatment outcomes will not be assessed as study outcomes.

All outcome indicators will be mapped to the domains of the RE-AIM framework, including reach, screening coverage and participant characteristics; effectiveness, LTBI positivity and agreement between reading methods; adoption, health worker participation and app use; implementation, data completeness, operational efficiency, and workflow fidelity; and maintenance, documentation of referral advice and potential integration into routine program activities.

### Data Analysis

Data analysis will be conducted using a combination of descriptive, inferential, and qualitative analytical approaches to evaluate screening outcomes and implementation processes. Quantitative data collected through the TBConnect app will be exported and compiled for analysis. Data cleaning, verification, and preliminary organization will be performed in Microsoft Excel, and records will be checked for completeness, consistency, and missing values before statistical analysis. Descriptive statistics will be used to summarize participant characteristics and screening indicators. Variables such as age, sex, exposure characteristics, screening pathway, and CyTB test results will be summarized using frequencies, proportions, means, and SDs where appropriate. Pathway-level analyses will focus on implementation performance rather than diagnostic superiority or causal effectiveness. Descriptive comparisons will be conducted for screening coverage, completion of CyTB administration, completion of 48-to-72-hour reading, LTBI positivity, completeness of digital records, time required for test administration and reading, documentation of referral advice, participant acceptability, health care worker acceptability, and operational challenges. Where data permit, pathway comparisons will be stratified or adjusted for relevant participant and contextual characteristics, including age, sex, relationship to the index tuberculosis patient, exposure history, distance from the health facility, and health facility catchment area. Findings will be interpreted in the context of each pathway’s operational context. Key screening outcomes, including the proportion of eligible HHCs screened and LTBI positivity among screened participants, will be calculated. Screening coverage will be determined as the proportion of identified HHCs who complete CyTB testing and follow-up reading. These statistical analyses will be conducted using SPSS software.

Agreement between app-assisted and manual induration measurements will be assessed in the validation subset using both continuous and categorical approaches. For continuous induration values recorded in millimeters, agreement will be assessed using the intraclass correlation coefficient, mean difference between methods, and Bland-Altman analysis with 95% limits of agreement. For categorical classification using the predefined ≥5 mm threshold, percentage agreement and Cohen κ will be calculated. Sensitivity, specificity, positive predictive value, and negative predictive value of app-assisted classification may also be estimated using manual classification as the reference standard. Associations between LTBI positivity and selected participant characteristics will be examined using chi-square tests for categorical variables. Variables identified as relevant will be included in multivariable logistic regression models to identify factors independently associated with LTBI positivity. Implementation and operational indicators such as health care worker proficiency, screening completion rates, follow-up reading rates, data completeness, and operational efficiency will also be analyzed using descriptive statistics.

Qualitative interview data will be analyzed using thematic analysis. Transcripts will be imported into NVivo, coded inductively, and grouped into categories and higher-order themes reflecting barriers and facilitators to implementation. Initial open coding will be conducted to identify meaningful segments of text related to participants’ experiences, the usability of the TBConnect app, the acceptability of CyTB screening, and operational challenges. Codes will then be grouped into categories and organized into broader themes reflecting barriers and facilitators to implementation.

### Quality Assurance

Health care workers will undergo structured training followed by assessment and certification before field deployment to support implementation fidelity and data quality. Pilot-testing will be undertaken to assess feasibility and refine workflow, screening procedures, and app use. During implementation, digital records and operational logs will be reviewed regularly to monitor completeness, consistency, and operational challenges. The logic model will guide optimization during the pilot phase, and the RE-AIM framework will guide the final evaluation.

### Ethical Considerations

Ethical approval was obtained from the Institutional Ethics Committee of ICMR-NIIRNCD (Indian Council of Medical Research–National Institute for Implementation Research on Non-Communicable Diseases), Jodhpur, Rajasthan, India (CD/TB/INTR-IM-2024-00135 on August 7, 2025). Written informed consent will be obtained from all adult participants. For participants younger than 18 years, written parental or guardian consent and age-appropriate assent will be obtained, as applicable.

Participant confidentiality will be maintained by assigning unique study identification numbers and storing all data collected via the TBConnect app on secure, password-protected systems with role-based access controls. Only authorized members of the research team will have access to identifiable information, and study findings will be reported in aggregate form. No monetary compensation will be provided to participants for participation in this study.

## Results

Ethical approval was obtained from the Institutional Ethics Committee of ICMR-NIIRNCD, Jodhpur, Rajasthan, India, on August 7, 2025. Further, the study is funded by the Indian Council of Medical Research, New Delhi. The study is planned for implementation over 24 months in the Balesar block, Jodhpur district, Rajasthan.

Participant recruitment and CyTB screening are projected to begin in December 2025 and end in December 2026. Data cleaning and statistical analysis are scheduled for after field data collection. The main findings are expected to be submitted for publication in May 2027.

## Discussion

### Principal Findings

This study is expected to provide implementation evidence on the use of a digitally supported CyTB-based LTBI screening model for HHCs of confirmed tuberculosis patients within routine primary health care services. Its main contribution will be to examine whether CyTB testing can be integrated with the TBConnect app to support key program processes, including participant registration, screening workflow documentation, induration recording, follow-up reading within 48-72 hours, and reporting. In addition to estimating LTBI positivity, the study will assess HHC coverage, completion of induration reading, data completeness, operational feasibility, and acceptability among participants and health care workers.

The comparison of health facility–based, home-based, and camp-based screening pathways aims to identify the most feasible approach to reaching and screening HHCs under routine program conditions. These pathways will be assessed as alternative delivery strategies for the same CyTB-TBConnect screening model, rather than as separate diagnostic interventions. The pathway showing the most favorable combination of HHC coverage, participant attendance, completion of 48-to-72-hour reading, data completeness, and acceptability may guide subsequent implementation within the study area.

The study will also assess agreement between app-assisted and manual induration measurements using paired observations recorded in TBConnect. This component is expected to provide field-level evidence on whether app-supported induration documentation can be used reliably within routine screening workflows.

HHCs of individuals with tuberculosis are an important group for LTBI screening because of their close exposure to infectious disease and increased risk of progression to active tuberculosis. This aligns with the prevention-oriented priorities of the NTEP, which emphasizes early identification and management of individuals at risk of tuberculosis [15]. Although LTBI testing is recognized as an important component of tuberculosis prevention, its implementation within routine public health services remains constrained by operational barriers, including participant mobilization, return visits for skin test reading, documentation requirements, and program-level monitoring [16].

CyTB offers a practical, skin-test–based approach that can be delivered in primary health care and community settings. When combined with a digital workflow, it may support more standardized documentation, follow-up tracking, induration recording, and reporting. However, evidence on the combined implementation of CyTB testing and an Android-supported workflow under routine program conditions in India remains limited. By evaluating this model through 3 service delivery pathways, the study is positioned to generate program-relevant evidence on implementation feasibility rather than focusing only on diagnostic performance [20].

A key strength of this study is its implementation within routine public health services using existing primary health care structures and frontline health care workers. This increases the relevance of the findings for program planning and future implementation. The use of a logic model during development and optimization, followed by RE-AIM–guided evaluation, provides a structured approach for assessing implementation outcomes. The mixed methods design will allow quantitative indicators of screening performance to be interpreted alongside qualitative findings on user experience, acceptability, and operational challenges. The validation of app-assisted induration measurement against manual measurement further strengthens the assessment of the digital workflow.

The study is being conducted in a single implementation setting, and findings may not be directly generalizable to other districts or health system contexts without further evaluation. The projected sample is based on the estimated number of eligible HHCs in the selected block. Still, the actual screening coverage will depend on consent rates, participant availability, and completion of the 48-to-72-hour reading. The study evaluates the implementation of screening and the documentation of referral advice; it does not assess the postreferral cascade, including clinical evaluation, initiation of tuberculosis preventive therapy, adherence, or treatment outcomes. Therefore, the study conclusions will be limited to the implementation and performance of the block-level CyTB-TBConnect screening model for LTBI.

The findings may help refine CyTB-based LTBI screening within routine primary health care services. Identification of a feasible and implementable delivery pathway may improve HHC coverage in the study area and guide further evaluation in other program settings. Evidence from the TBConnect workflow may also inform digital documentation, follow-up tracking, and induration recording for LTBI screening. Study findings will be disseminated through peer-reviewed publications, scientific meetings, and communication with stakeholders in district and state tuberculosis programs.

### Conclusions

This protocol describes the implementation and evaluation of a digitally supported CyTB-based LTBI screening model for HHCs of confirmed tuberculosis patients in Rajasthan, India. By assessing screening coverage, completion of 48-to-72-hour induration readings, pathway feasibility, agreement between app-assisted and manual induration readings, digital data completeness, and user acceptability, the study will generate operational evidence relevant to LTBI screening within routine primary health care services. The findings may help refine HHC screening approaches and guide further evaluation of CyTB-TBConnect-supported screening models in other program settings.

## Abbreviations

BCG: Bacillus Calmette-Guérin
CHC: community health center
HHC: household contact
ICMR-NIIRNCD: Indian Council of Medical Research–National Institute for Implementation Research on Non-Communicable Diseases
IGRA: interferon-gamma release assay
LTBI: latent tuberculosis infection
NTEP: National Tuberculosis Elimination Programme
PHC: primary health center
RE-AIM: reach, effectiveness, adoption, implementation, and maintenance
TST: tuberculin skin test
